# MERS Coronaviruses in Dromedary Camels, Egypt

**DOI:** 10.3201/eid2006.140299

**Published:** 2014-06

**Authors:** Daniel K.W. Chu, Leo L.M. Poon, Mokhtar M. Gomaa, Mahmoud M. Shehata, Ranawaka A.P.M. Perera, Dina Abu Zeid, Amira S. El Rifay, Lewis Y. Siu, Yi Guan, Richard J. Webby, Mohamed A. Ali, Malik Peiris, Ghazi Kayali

**Affiliations:** The University of Hong Kong, Hong Kong, China (D.K.W. Chu, L.L.M. Poon, R.A.P.M. Perera, Y. Guan, M. Peiris);; National Research Centre, Giza, Egypt (M.M. Gomaa, M.M. Shehata, D.A. Zeid, A.S. El Rifay, M.A. Ali);; HKU-Pasteur Research Pole, Hong Kong (L.Y. Siu);; St. Jude Children’s Research Hospital, Memphis, Tennessee, USA (R.J. Webby, G. Kayali)

**Keywords:** zoonosis, pneumonia, genomics, phylogeny, dromedary, camel, Egypt, viruses, coronaviruses, Middle East respiratory syndrome, MERS, MERS-CoV

## Abstract

We identified the near-full-genome sequence (29,908 nt, >99%) of Middle East respiratory syndrome coronavirus (MERS-CoV) from a nasal swab specimen from a dromedary camel in Egypt. We found that viruses genetically very similar to human MERS-CoV are infecting dromedaries beyond the Arabian Peninsula, where human MERS-CoV infections have not yet been detected.

Middle East respiratory syndrome (MERS) is a pneumonic illness caused by a novel lineage C betacoronavirus (CoV). During September 2012–January 20, 2014, a total of 178 confirmed cases in humans resulted in 76 deaths (*1*). Primary infections have originated from countries within the Arabian Peninsula, although travel-associated cases and some secondary transmission have been reported in other countries. Limited human-to-human transmission has resulted in clusters of cases, some associated with multiple rounds of human-to-human transmission (*2*); the remaining sporadic cases in humans are presumed to be of zoonotic origin.

MERS-CoV genomes are phylogenetically classified into 2 clades, clade A and B clusters (*3*). The viral genomes detected in the earliest cases in humans (clade A cluster; EMC/2012 and Jordan-N3/2012) are genetically distinct from the others (i.e., clade B). The proximate source of human infection remains unclear. MERS-CoV is related to, but not identical to, viruses detected in bats (*4*,*5*). A short RNA fragment of the conserved viral polymerase region identical to MERS-CoV has been identified in *Taphozous perforates* bats, but these findings need to be confirmed (*6*).

Serologic studies of domestic livestock in Jordan, Saudi Arabia, Qatar, United Arab Emirates, and Egypt have found high seroprevalance to a MERS-like CoV in dromedary camels but not in other domestic animals (*7*–*11*). An investigation of domestic animals in the vicinity of 2 persons with related infections detected fragments of viral RNA in dromedary camels in contact with these persons but whether this represented transmission from a unidentified source to humans and dromedaries, transmission from humans to dromedaries, or vice versa is not clear (*12*).

## The Study

We collected nasal swab specimens from 110 apparently healthy dromedaries (*Camelus dromedarius*) >6 years of age in abattoirs on 12 occasions during June–December 2013 (Table 1). Serum was collected from 52 of these dromedaries. Serum from 179 persons working in the camel abattoirs also was collected. Median age of these workers was 38 years (range 9–67 years), 84% were male, and 25 reported comorbidities (i.e., cardiovascular, renal, diabetes, other). Collection of the human specimens was approved by the ethics committee of the National Research Centre (Giza, Egypt), and Institutional Animal Care and Use Committee approval for collection of animal samples was obtained from St Jude Children’s Research Hospital (Memphis, TN, USA).

**Table 1:** Results of testing nasal swab specimens from dromedary camels by RT-PCR for MERS-CoV and for other CoVs, Egypt, 2013*

Location of animals sampled	No. samples tested	No. MERS-CoV positive†	No. other CoV positive. (virus identified)
Alexandria‡	17	0	0
Cairo			
Abattoir 1§	46	0	3 (BCoV)
Abattoir 2§	10	3	0
Nile Delta region, abattoir§	37	1	5 (BCoV)

*RT-PCR, reverse transcription PCR; MERS, Middle East respiratory syndrome; CoV, coronavirus; BCoV, bovine coronavirus. Nasal swabs from dromedary camels were placed in phosphate-buffered saline/glycerol transport medium, kept on ice during the field trip and stored at −80°C on arriving at the laboratory. Specimens were shipped in dry ice to the University of Hong Kong laboratory, where they were stored at −80°C until testing. †Taken as positive only when both upstream of E gene and open reading frame 1a RT-PCR were positive. ‡Locally reared animals. §Imported from Sudan or Ethiopia.

Real-time reverse transcription PCR (RT-PCR) targeting upstream of E gene of MERS-CoV was used for screening. The open reading frame (ORF) 1a gene was used for confirmation as recommended by the World Health Organization(www.who.int/csr/disease/coronavirus_infections/MERS_Lab_recos_16_Sept_2013.pdf?ua = 1). We also used a previously described pan-CoV nested PCR targeting the viral RNA-dependent RNA polymerase (RdRp) region (*13*), and PCR products were analyzed by sequencing (Technical Appendix).

We detected MERS-CoV RNA in 4 (3.6%) of 110 nasal swab specimens from dromedary camels with the upstream of E gene assay (cycle threshold [C_t_] 23.2–36.8), confirmed by the ORF1a assay (C_t_ 23.2–39.1), fulfilling the World Health Organization criteria for diagnosis of MERS-CoV infection (Table 1). PCR was repeated from a fresh RNA extract to confirm positive results. One positive sample was obtained from a camel in an abattoir in the Nile Delta region in November 2013 and 3 other samples from an abattoir in Cairo in December 2013. Virus culture attempts in Vero E6 cells (ATCC CRL-1586) so far have been unsuccessful. The pan-CoV nested PCR detected CoV in an additional 8 specimens from dromedary camels. Sequence analyses of these additional positive samples showed that these amplicon sequences were genetically similar to those of bovine coronavirus (BCoV) (>99% nucleotide similarity). No animal was co-infected with MERS-CoV and BCoV-like viruses. The animals positive for either MERS-CoV or BCoV-like virus were all imported from Sudan or Ethiopia for slaughter.

**Figure 1:**
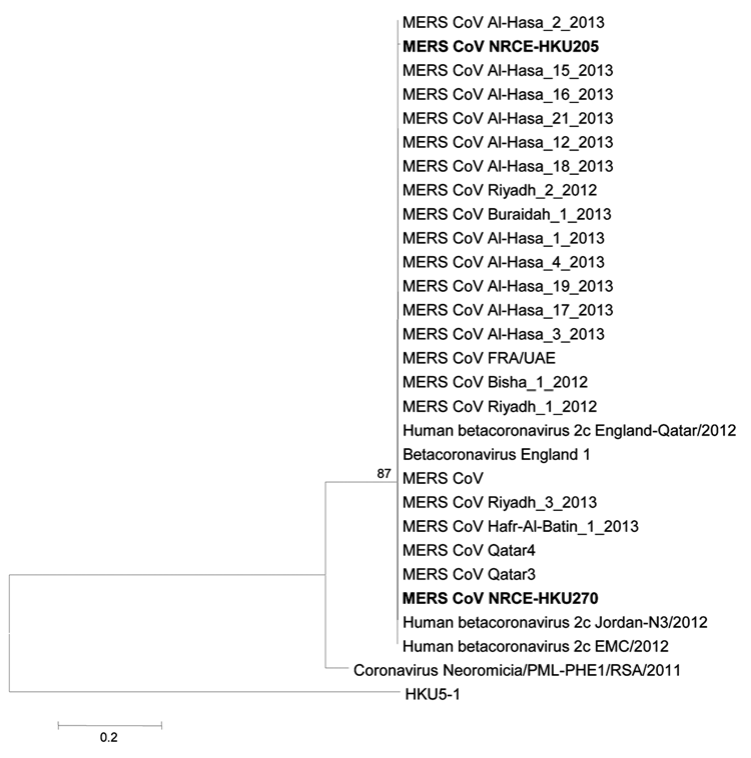
Phylogenetic analyses of a partial RNA-dependent RNA polymerase (RdRp) sequence determined from samples from dromedary camels (*Camelus dromedarius*) NRCE-HKU205 and NRCE-HKU270 that were positive for Middle East respiratory syndrome coronavirus (MERS-CoV). The viral RdRp region analyzed is a highly conserved region of the genome (covering motif B of RdRp) in nonstructural protein 12, at position 15202–15582 of MERS-CoV genome. The partial RdRp sequence of NRCE-HKU205 (GenBank accession no. KJ477102) and NRCE-HKU270 (GenBank accession no. KJ477103) was aligned with human MERS-CoVs (GenBank accession nos. KF600652, KF600630, KF600651, KF186567, KF600627, KF186564, KF600634, KF600632, KF600644, KF600647, KF600645, KF186565, KF186566, KF745068, KF600620, KF600612, KC667074, KC164505, KF192507, KF600613, KF600628, KF961222, KF961221, KC776174, and JX869059) and other representative animal betacoroanviruses (GenBank accession nos. HKU5–1, EF065509; BtCoV/PML/Neo cf. zul/RSA/2011, KC869678). Bat CoV HKU5–1 and bat CoV/PML/Neo cf. zul/RSA/2011 were included in the analysis as outgroups because they are phylogenetically closest to MERS-CoV. Phylogenetic trees were constructed by using MEGA5 (*14*) with neighbor-joining method. Numbers at nodes indicate bootstrap values determined by 500 replicates. Only bootstrap values >70 are denoted. Bold type indicates MERS-CoV identified in the current study. Scale bars indicate the estimated genetic distance of these viruses.

On phylogenetic analysis, the partial RdRp sequences from MERS-CoV–positive samples NRCE-HKU205 and NRCE-HKU270 grouped within the cluster of MERS-CoV (Figure 1). The viral load in the other 2 specimens was too low to provide amplicons suitable for genetic sequencing. Viral RNA from NRCE-HKU205, the first positive specimen detected, was selected for more detailed genomic sequencing. We amplified viral RNA by PCR, using primers specific for overlapping regions of human MERS-CoV genome. PCR products were sequenced and assembled to produce a near full-length genome, lacking only the 3′ untranslated region (29908 nt, >99% of the MERS-CoV genome) (online Technical Appendix). The camel MERS-CoV genome has an overall nucleotide similarity of 99.2%−99.5% and deduced amino acid similarity of 98.0%–100% to ORFs of human MERS-CoV EMC/2012 (Table 2). The 5′ untranslated region and internal transcriptional regulatory sequences of NRCE-HKU205 were identical to published human MERS-CoV sequences.

**Table 2:** Percentage identity between ORFs of dromedary camel MERS-CoV (NRCE-HKU205) and human MERS-CoV (EMC/2012) at the nucleotide and amino acid levels*

ORF	% Identity to HCoV-EMC/2012	
	Nucleotide	Amino acid
ORF1a	99.5	99.2
ORF1b	99.5	99.7
S	99.2	98.9
ORF3	99.0	98.0
ORF4a	99.0	100
ORF4b	99.4	99.1
ORF5	99.4	98.6
E	100	100
M	100	100
N	99.4	99.2
ORF8b	99.1	98.2

*ORF, open reading frame; MERS, Middle East respiratory syndrome; CoV, coronavirus; H, human; E, envelope; M, membrane; N, nucleocapsid.

Using MERS-CoV EMC/2012 as a reference sequence, we found 12 aa differences (residues 23, 26, 194, 434, 666, 696, 756, 886, 888, 918, 1158, and 1333 of EMC/2012) in the spike protein of dromedary MERS-CoV NRCE-HKU205 and 1 aa deletion (residue 1293 of EMC/2012) in the N terminal of the transmembrane domain. NRCE-HKU270 virus spike does not have a deletion of residue 1293. Of these, only residue 434 falls within the proposed receptor binding domain of spike protein, but it is located at the core (stem) subdomain of the receptor binding domain, suggesting that the camel MERS-CoV is still likely to bind human CD26 (Technical Appendix Figure). The biological impact of this difference and other amino acid differences needs to be fully explored.

**Figure 2:**
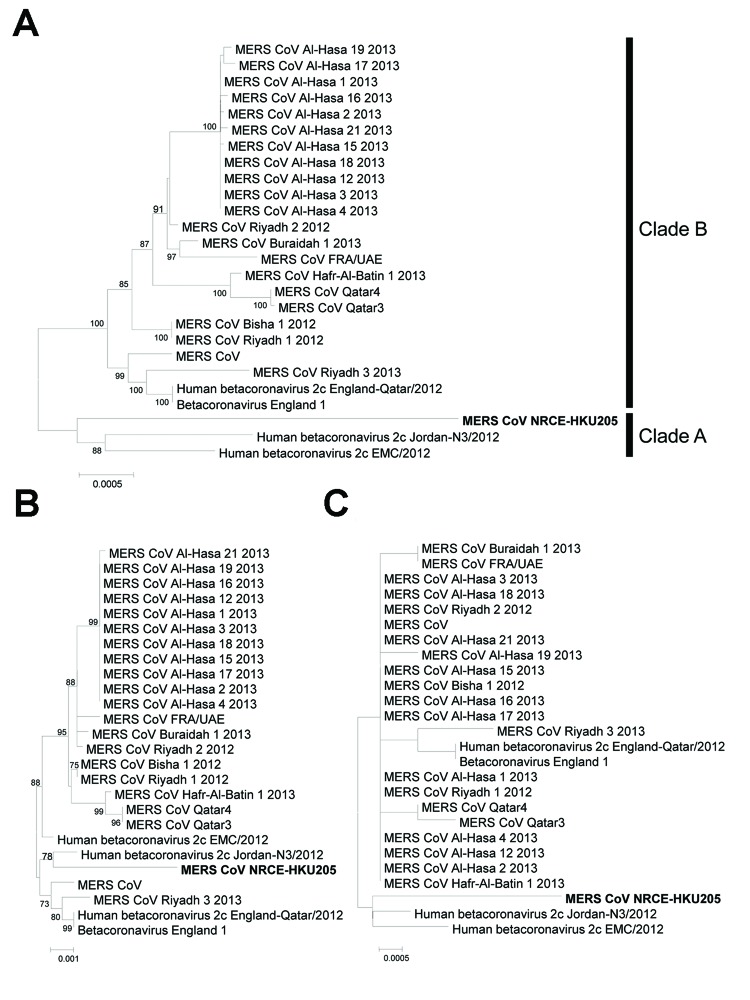
Phylogenetic analyses of Middle East respiratory syndrome coronavirus (MERS-CoV) from dromedary camels. Genomic (A), spike (B), and nucleocapsid (C) sequences of the dromedary camel MERS-CoV NRCE-HKU205 (GenBank accession no. KJ477102) were aligned with the corresponding human MERS-CoV (N = 25) sequences retrieved from GenBank (accession nos. as in Figure 1 legend). Phylogenetic trees were constructed by using MEGA5 (*14*) with neighbor-joining method. Numbers at nodes indicate bootstrap values determined by 500 replicates. Only bootstrap values >70 are denoted. Bold type indicates MERS-CoV identified in the current study. Scale bars indicate the estimated genetic distance of these viruses.

We used phylogenetic analyses of the full genome, the spike gene, and nucleocapsid gene of MERS-CoV NRCE-HKU205 to compare with the same genes of other human MERS-CoVs (Figure 2). NRCE-HKU205 is within the clade A group but distinct from MERS-CoV EMC/2012, the only other MERS-CoV isolate available in our laboratory, excluding laboratory cross-contamination as an explanation for the detection of MERS-CoV from the dromedary specimens.

Using a previously described pseudoparticle neutralization assay (*8*), we detected antibodies against MERS-CoV (titer >20) in 48 (92.3%) of 52 dromedary serum samples with titers ranging from 20 to >640. Dromedary NRCE-HKU205 had a serum antibody titer of 640, possibly indicating a developing serologic response, as was noted previously (*7*), or the possibility of re-infection. Serum was not available from the other 3 animals with MERS-CoV RNA-positive nasal swab specimens.

Serum from 179 persons working in the dromedary abattoirs was negative for antibody to MERS-CoV. This finding includes 114 persons working in the 2 abattoirs from which the MERS-CoV–positive animal swab specimens were obtained.

## Conclusions

Our findings confirm that MERS-CoV infects dromedary camels and that this virus is genetically very similar to a MERS-CoV that is infecting humans. The detection of MERS-CoV in nasal swab specimens of camels in 2 of 12 sampling occasions in abattoirs, taken together with the high seropositivity to MERS-CoV in dromedaries previously reported, supports the contention that MERS-CoV infection is common in dromedaries. Studies of dromedaries within camel herds and through the animal marketing system supplying abattoirs are needed to define the epidemiology of the infection. Our findings strengthen the plausibility that dromedaries may be a potential source of human infection and emphasize the need for detailed epidemiologic investigation of the exposure histories of humans with MERS. However, the lack of serologic evidence of infection of humans working in these abattoirs suggests that transmission of this virus to humans is uncommon. The detection of MERS-CoV in dromedaries in Egypt, in animals imported from Sudan and Ethiopia, suggests that cases may occur in humans beyond the Arabian Peninsula. MERS CoV diagnostic tests should be considered for all patients with unexplained severe pneumonia in Egypt, northeastern Africa, and beyond.

Technical Appendix The sequencing method and the structure of Middle East respiratory syndrome coronavirus spike protein receptor binding domain and receptor protein complex.
