# Supplementary material for: MERS Coronaviruses in Dromedary Camels, Egypt
Source: Emerg Infect Dis. 2014 Jun;20(6):1049–53. doi: 10.3201/eid2006.140299 (PMC4036765; doi:10.3201/eid2006.140299)
Supplement: Technical Appendix — The sequencing method and the structure of Middle East respiratory syndrome coronavirus spike protein receptor binding domain and receptor protein complex. [file 14-0299-Techapp-s1.pdf]

Article DOI: http://dx.doi.org/10.3201/eid2006.140299

MERS Coronaviruses in Dromedary Camels, Egypt

Technical Appendix

The sequencing method was as follows: randomly primed cDNA products of the camel MERS-CoV were amplified into short DNA fragments (≈500 bp) by nested PCRs. Primers were designed on the basis of human MERS-CoVs (available on request). Overlapping PCR products were sequenced with forward and reverse primers used in the corresponding nested PCR amplification. The sequences (without primer sequences) were aligned and assembled by BioEdit (www.mbio.ncsu.edu/bioedit/page2.html). The genome was sequenced ≥2 times.

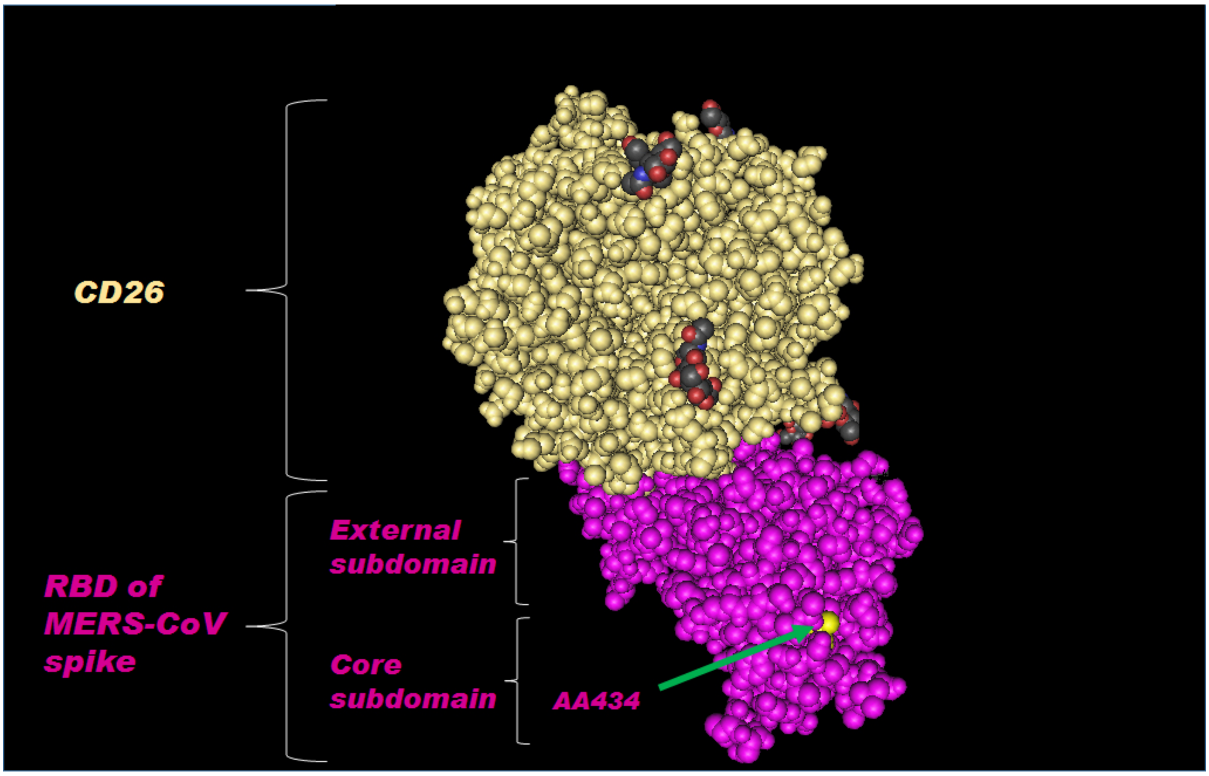

Technical Appendix Figure. Structure of Middle East respiratory syndrome coronavirus (MERS-CoV) spike protein receptor binding domain (RBD) (purple) and CD26 (blond) receptor protein complex. The structure shown is modified from a published structure (PBD: 4KR0). The external subdomain of the spike protein which is responsible for binding to the receptor CD26 and the core subdomain of RBD are shown. The amino acid position which differs between dromedary camel MERS-CoV NRCE-HKU205 (AA 434S) and human MERS-CoV EMC/2012 (AA 434A) is highlighted by an arrow.
